# Genomic and transcriptomic profiling of phoenix colonies

**DOI:** 10.1038/s41598-022-18059-1

**Published:** 2022-08-12

**Authors:** Devin Sindeldecker, Matthew Dunn, Aubree Zimmer, Matthew Anderson, Juan Alfonzo, Paul Stoodley

**Affiliations:** 1 Department of Microbial Infection and Immunity, The Ohio State University, 760 BRT, 460 West, 12th Avenue, Columbus, OH 43210 USA; 2 Department of Microbiology, The Ohio State University, Columbus, OH USA; 3 Ohio State Biochemistry Program, The Ohio State University, Columbus, OH USA; 4 Department of Orthopaedics, The Ohio State University, Columbus, OH USA; 5 National Center for Advanced Tribology at Southampton (nCATS), Mechanical Engineering, University of Southampton, Southampton, UK

**Keywords:** Antimicrobial resistance, Gene expression

## Abstract

*Pseudomonas aeruginosa* is a Gram-negative bacterium responsible for numerous human infections. Previously, novel antibiotic tolerant variants known as phoenix colonies as well as variants similar to viable but non-culturable (VBNC) colonies were identified in response to high concentrations of aminoglycosides. In this study, the mechanisms behind phoenix colony and VBNC-like colony emergence were further explored using both whole genome sequencing and RNA sequencing. Phoenix colonies were found to have a single nucleotide polymorphism (SNP) in the PA4673 gene, which is predicted to encode a GTP-binding protein. No SNPs were identified within VBNC-like colonies compared to the founder population. RNA sequencing did not detect change in expression of PA4673 but revealed multiple differentially expressed genes that may play a role in phoenix colony emergence. One of these differentially expressed genes, PA3626, encodes for a tRNA pseudouridine synthase which when knocked out led to a complete lack of phoenix colonies. Although not immediately clear whether the identified genes in this study may have interactions which have not yet been recognized, they may contribute to the understanding of how phoenix colonies are able to emerge and survive in the presence of antibiotic exposure.

## Introduction

*Pseudomonas aeruginosa* is a Gram-negative bacterium found throughout the natural environment. As an opportunistic pathogen, it is most commonly associated with cystic fibrosis (CF) infections but can also reside in chronic wounds and post-surgical site infections^1–3^. *P. aeruginosa* utilizes the formation of biofilms, persister cells, and development of multidrug resistance mechanisms to evade killing by antimicrobial agents^4–7^. In addition to these antimicrobial tolerance and resistance mechanisms, *P. aeruginosa* has been found to rapidly adapt to its environment within the context of infection, leading to concerns of reduced effectiveness of antimicrobial agents in eradicating *P. aeruginosa*^8^.

In a previous study, our lab identified a novel, aminoglycoside tolerant phenotype of *P. aeruginosa*, which we have termed phoenix colonies. Phoenix colonies are able to survive and thrive in an antibiotic laden environment. However, once removed from the initial antibiotic environment, phoenix colonies revert to a wild-type level of antibiotic susceptibility^9^. Phoenix colonies differ from traditional tolerance in that they are able to maintain high levels of metabolic activity throughout antibiotic exposure, whereas traditional tolerance is typically the result of slow growth or a decrease in metabolism. Additionally, phoenix colonies are distinct from both heteroresistant colonies and persister cells. Using population analysis profiling, a traditional method for identifying heteroresistance, phoenix colonies were found to be homogenously antibiotic susceptible after isolation. The tobramycin concentration in the region of phoenix colony emergence was also measured to be approximately ten times the minimum inhibitory concentration (MIC), which would prevent persister cell reawakening at the time when phoenix colonies appear^10^. In the same study, we identified an additional phenotype which was similar to the viable but non-culturable colonies (VBNCs^11^) phenotype, however, these “VBNC-like” colonies were able to grow in the initial antibiotic environment but were unable to be cultured otherwise, including cultures containing the same antibiotic^9^. While the significance of these phenotypes in a clinical setting is currently unknown, it is possible that phoenix colonies or VBNC-like colonies could lead to chronic or recurrent infections, similar to persister cells^7^. Furthermore, while it appears that the phoenix colonies only emerge in the presence of aminoglycosides^9^, the molecular and genetic mechanisms leading to phoenix colony and VBNC-like emergence are unknown. Understanding the genetic alterations in these cells or alterations to gene expression may allow for better preventative or treatment options in the management of chronic or recurrent infections.

In this study, we used whole genome sequencing (WGS) of phoenix colonies and VBNC-like colonies to identify a single nucleotide polymorphism (SNP) which may be associated with the emergence of phoenix colonies in the presence of high concentrations of aminoglycosides. Additionally, RNA sequencing was performed on phoenix colonies as well as VBNC-like colonies and identified differentially expressed genes (DEGs) which could account for the antibiotic adaptation phenotypes portrayed by both phoenix colonies and VBNC-like colonies.

## Results

### Phoenix colonies contain a SNP in PA4673

**Figure 1:**
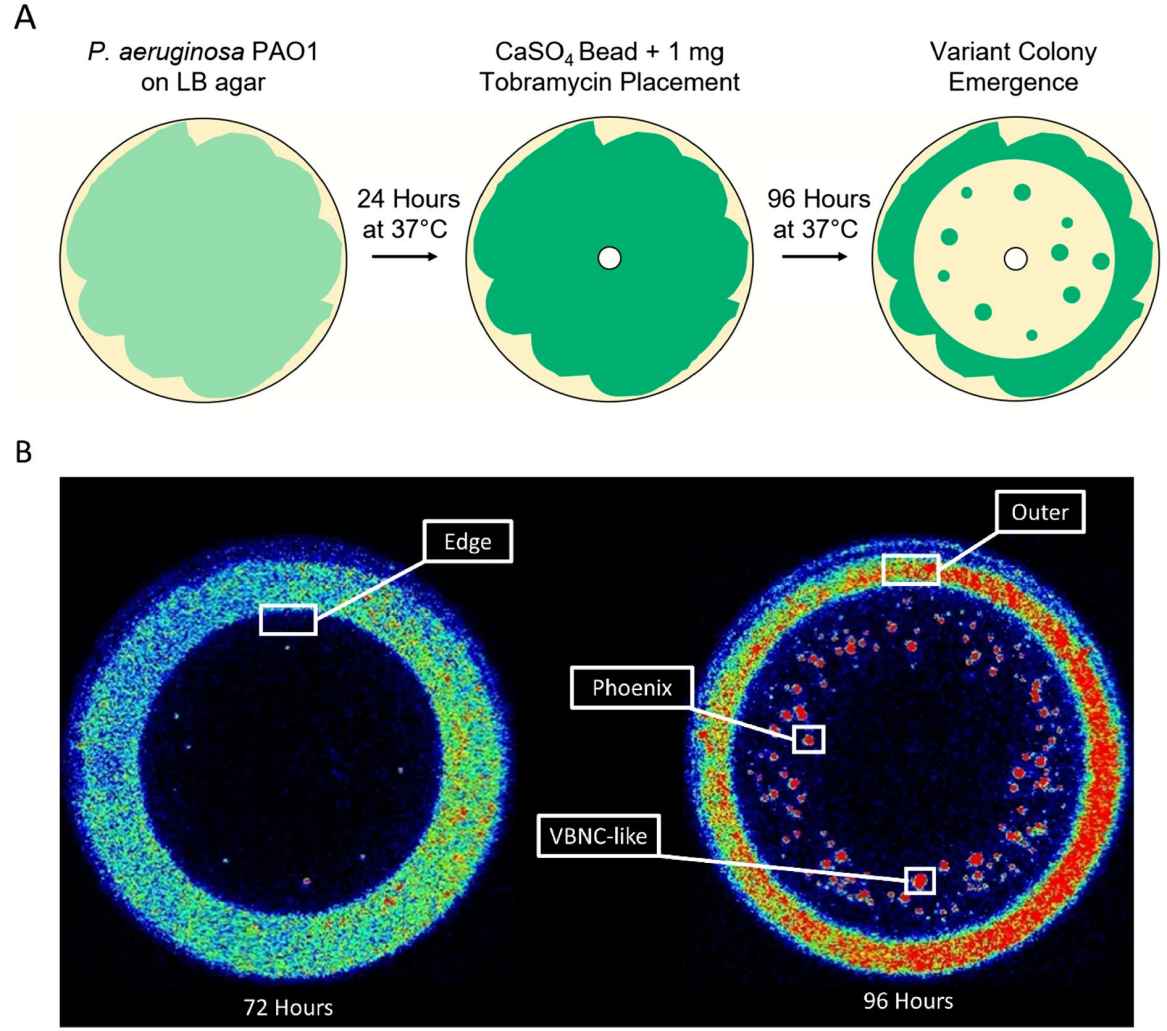
Isolation of *P. aeruginosa* colony variants for assaying the genome and gene expression. (**A**) Diagram of the experimental methods for obtaining variant colony emergence for *P. aeruginosa* PAO1 with tobramycin. (**B**) In vitro imaging system (IVIS) images of representative phoenix colony plates show areas of bacterial sampling. Seventy-two hours post tobramycin bead placement, samples were taken from the edge of the zone of clearance (ZOC) where phoenix colonies would be expected to arise the following day after continued incubation. Ninety-six hours post tobramycin bead placement, samples were taken from phoenix colonies, VBNC-like colonies, and the outer background lawn. Additionally, samples were taken from bacterial lawns grown for 72 h that had not been exposed to antibiotics to be used as comparative controls. Red indicates high levels of metabolic activity, blue indicates low levels of metabolic activity, black indicates no metabolic activity.

As shown previously^9^, antibiotic resistant and tolerant variant colonies were cultured by exposing a *P. aeruginosa* PAO1 biofilm lawn to high concentrations of tobramycin through exposure using a CaSO_4_ bead containing 1 mg of tobramycin (Fig. 1). Genomic DNA was isolated from phoenix colonies, VBNC-like colonies, and the founder population of *P. aeruginosa* PAO1. Whole genome sequencing (WGS) was performed using a Nanopore MinION Sequencer, and the reads were mapped to the PAO1 reference genome. Mapped reads were analyzed for the presence of SNPs as well as insertions or deletions (indels). Only the phoenix colony sample encoded any SNPs compared to the PAO1 reference genome and no samples (phoenix colony, VBNC-like colony, or control colony) contained indels. The single guanine to thymine (GCF_000006765.1:c.353G>T) variant occurred within the PA4673 gene, a hypothetical cytosolic protein, to produce a serine to isoleucine (S118I) amino acid change.

### PA4673 is predicted to encode a GTP-binding protein

**Figure 2:**
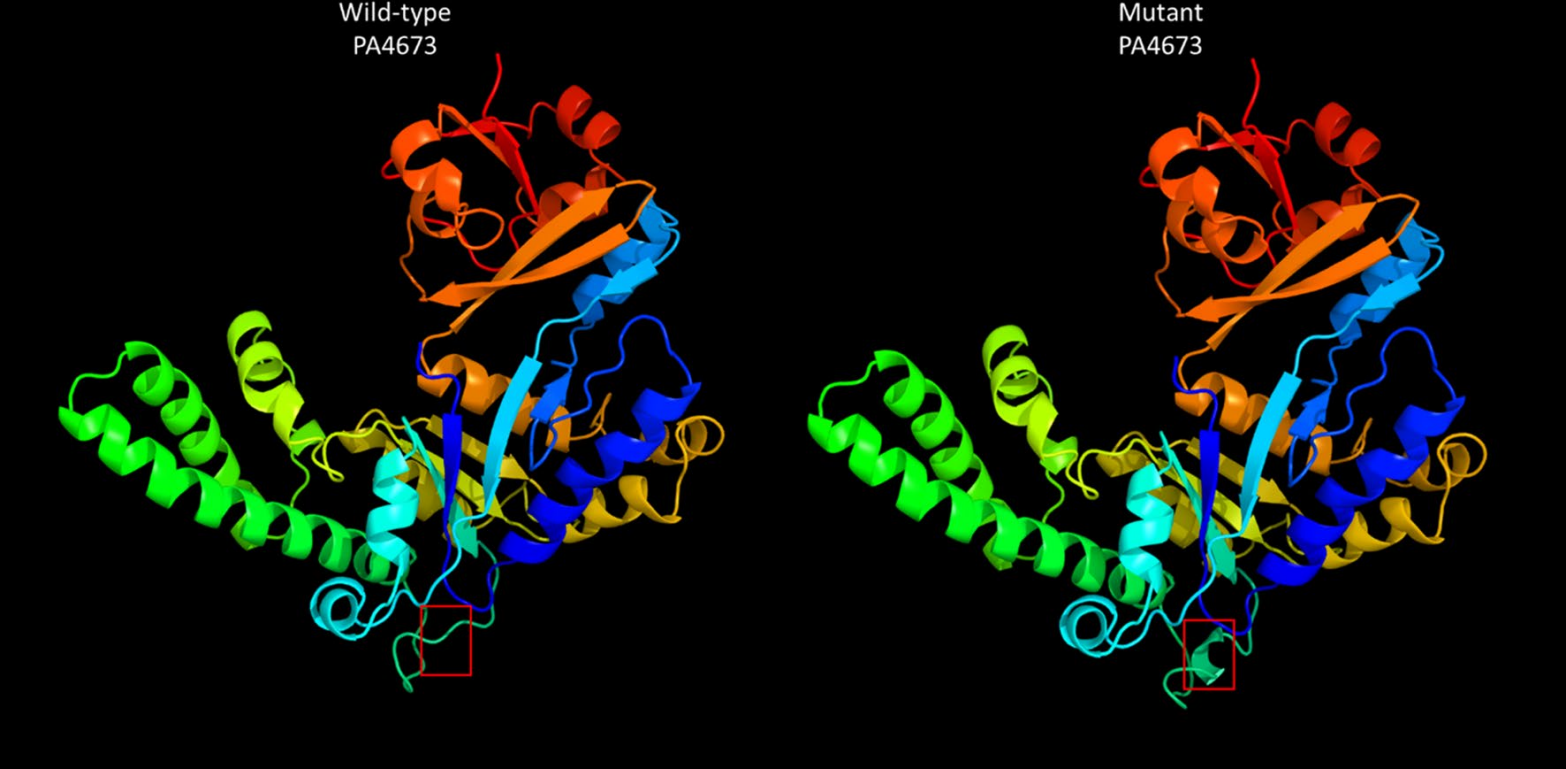
Protein structure of wild-type and mutant PA4673. Protein structures were derived from the amino acid sequences of wild-type and mutant PA4673 using Phyre2. The red box indicates the structural change caused by the SNP. Functional domain identification in both PA4673 isoforms indicates that they likely function as GTP-binding proteins. As the SNP causes an amino acid change in the exterior portion of the predicted protein structure, it may lead to alteration of a binding site or protein function.

To determine the impact of the S118I polymorphism in PA4673, de novo protein modeling was performed using Phyre2^12^. The structural models of both the wild-type and mutant proteins show a change in the secondary structure of the mutant protein, specifically, the formation of a predicted β sheet at the mutation site, which is located in an exposed face of the protein and is also predicted to have an altered hydrophobicity in the mutated region (Fig. 2). This modification may lead to alteration of a binding site or protein function. Identification of functional domains in both PA4673 isoforms predicted domains between AA001 and AA363, indicating they likely function as GTP-binding proteins as they align with 100% confidence to the YchF GTP-binding protein of *Schizosaccharomyces pombe*^13,14^. Additionally, the PA4673 transposon mutant from the Colin Manoil *P. aeruginosa* transposon mutant library^15^ was screened for phoenix colony emergence. No significant difference was observed between wild-type *P. aeruginosa* PAO1 and the PA4673 transposon mutant (p = 0.71).

### DEGs were identified for phoenix colonies and VBNC-like colonies

Due to the lack of clear genetic candidates for promoting alternative colony states, a transcriptomic approach was utilized to define gene regulation changes that are associated with each alternative colony type. RNA was isolated from samples at the edge of the ZOC at 72 h, from multiple colony populations at 96 h (phoenix colonies, VBNC-like colonies, and the outer background lawn), and from a 72 h unexposed lawn as a control, and prepared for Illumina-based sequencing (Fig. 1). Although phoenix colonies were isolated at 96 h post antibiotic exposure, they had only been growing for approximately 24 h as distinct colonies within the ZOC and were still metabolically active (Fig. 1), indicating that they were still actively employing methods to survive antibiotic exposure.

**Figure 3:**
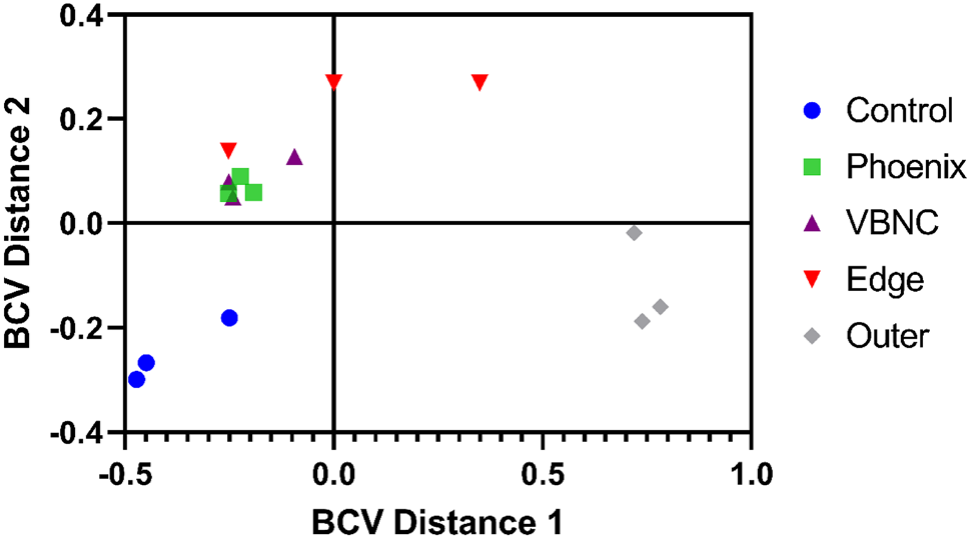
Multi-dimensional scaling (MDS) plot showing clustering of isolates. An MDS plot was generated using biological coefficients of variation (BCV) from transcript counts for each isolate sample. Samples from each group cluster well together aside from the Edge samples, and the phoenix colonies and VBNC-like colonies overlap considerably. Control lawn, Edge, and Outer samples cluster on their own and separately from the Phoenix and VBNC-like samples.

**Figure 4:**
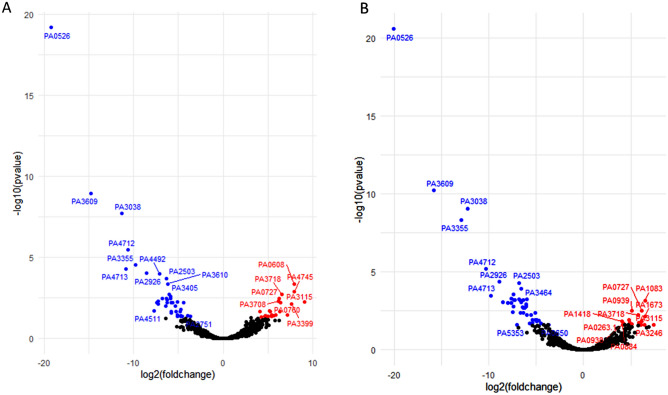
Volcano plots of DEGs. (**A**) Comparison of phoenix colony transcript counts to control lawn transcript counts showed 63 DEGs. (**B**) Comparison of VBNC-like colony transcript counts to control lawn transcript counts showed 90 DEGs. (**A**, **B**) Red dots indicate gene transcripts which were significantly upregulated (greater than twofold change and corrected p-value of 0.05 or less). Blue dots indicate gene transcripts which were significantly downregulated (greater than twofold change and corrected p-value of 0.05 or less). Black dots indicate genes with no-significant difference in transcription.

Differential gene expression of either phoenix colonies or VBNC-like colonies was performed to define transcriptional profiles that distinguish each phenotype. First, a multi-dimensional scaling (MDS) plot was generated using expression of all genes to examine the relationships between all samples (Fig. 3). This plot shows compact clustering among biological replicates of each colony phenotype with the exception of 96-h outer-edge populations, while the edge of ZOC group, control, and outer background lawn samples clustered on their own. The phoenix colonies and VBNC-like colonies overlap heavily, indicating highly similar transcriptomic profiles. Differential gene expression between the control lawn and phoenix colonies, as well as the control lawn and VBNC-like colonies, identified 63 and 90 genes with significantly different transcript abundance, respectively (> 2× fold change, corrected p value < 0.05, Fig. 4). These genes were split with 40 down-regulated and 23 up-regulated genes when comparing phoenix colonies to the control lawn, and 56 down-regulated and 34 up-regulated genes for VBNC-like colonies compared to the control (Tables 1, 2). There was no significant differential expression of PA4673, the gene containing the phoenix colony SNP. The DEGs were submitted for DAVID, KEGG, and Gene Ontology analysis, but no significant correlation was identified.

**Table 1:**
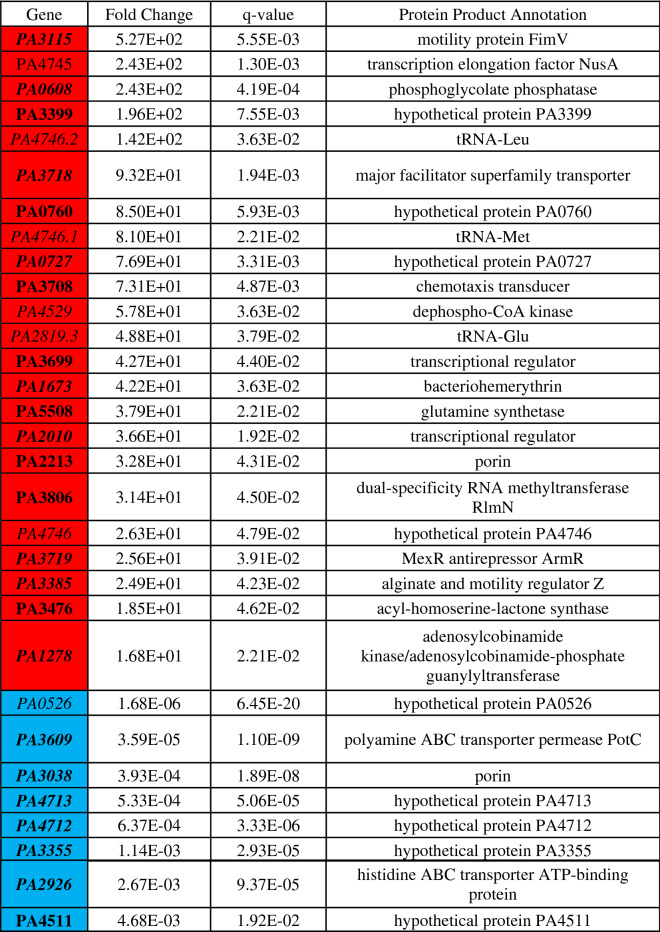

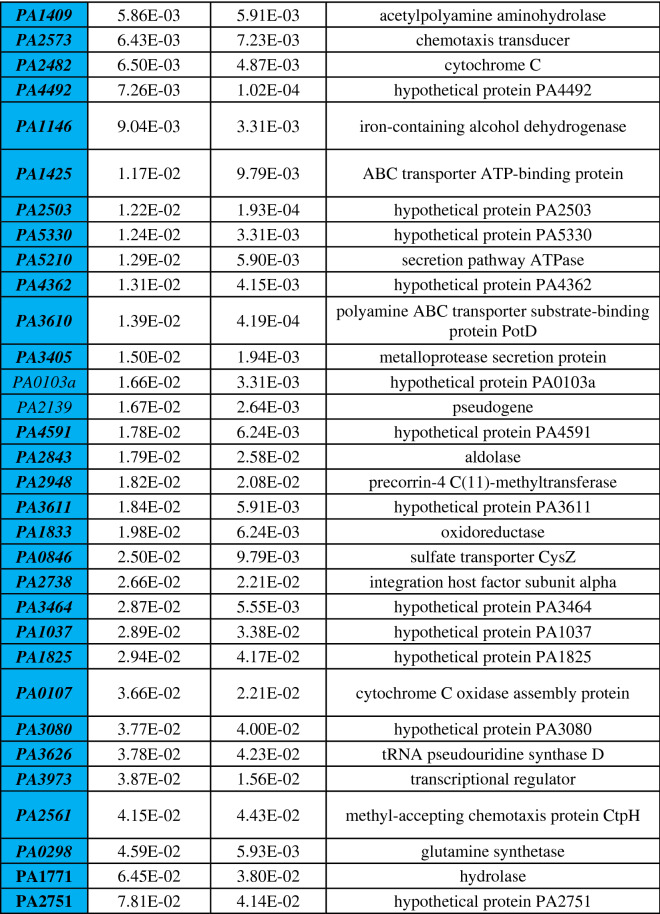
Statistically significant DEGs of phoenix colonies.

Phoenix colony transcripts were compared to the control lawn to identify genes which were significantly up or down regulated. Red indicates genes which were significantly upregulated and blue indicates genes which were significantly downregulated. Genes in bold were screened using transposon mutants. Genes in italics are shared by both phoenix colonies and VBNC-like colonies.

**Table 2:**
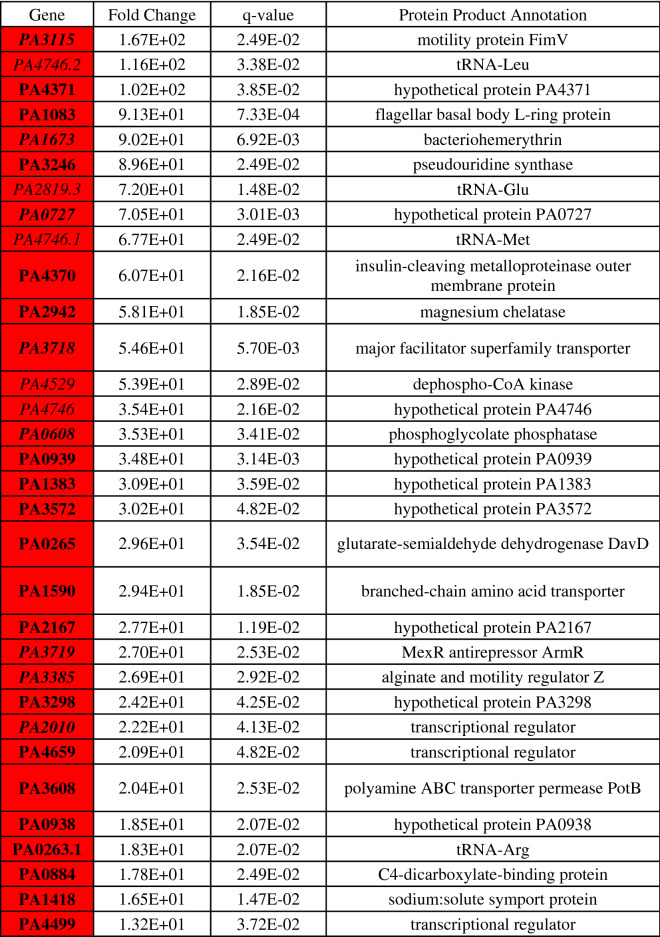

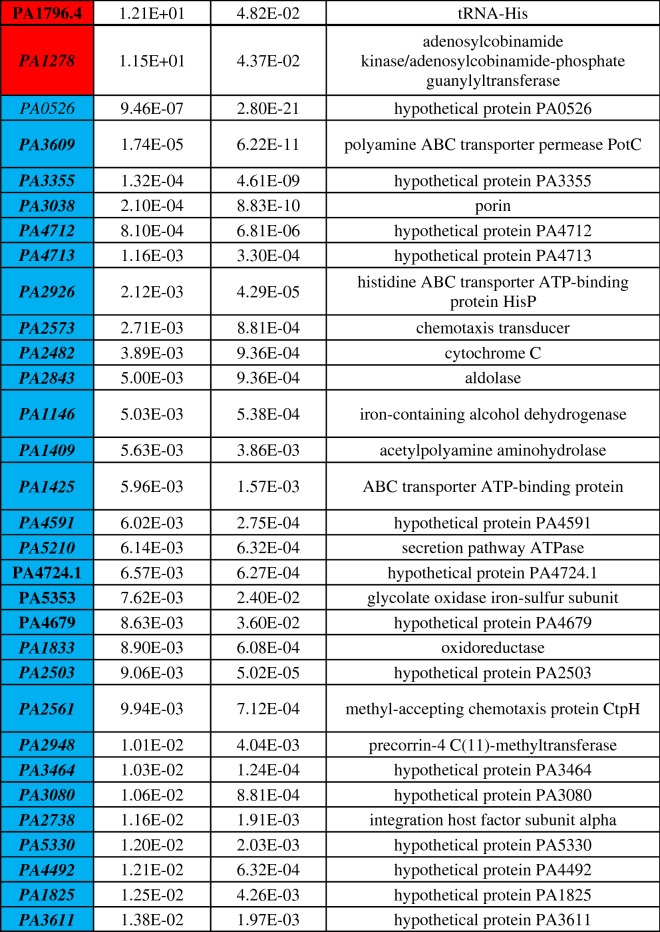

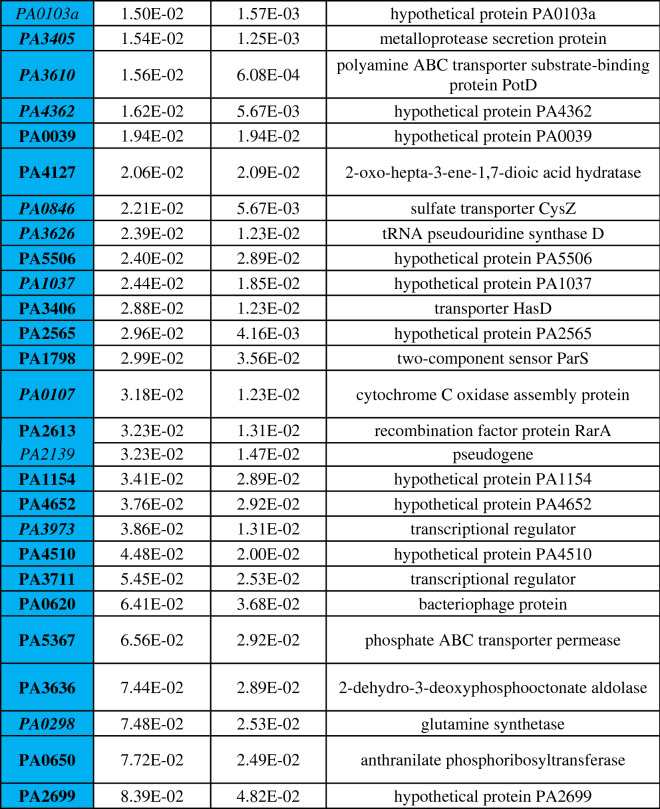
Statistically significant DEGs of VBNC-like colonies.

VBNC-like colony transcripts were compared to the control lawn to identify genes which were significantly up or down regulated. Red indicates genes which were significantly upregulated and blue indicates genes which were significantly downregulated. Genes in bold were screened using transposon mutants. Genes in italics are shared by both phoenix colonies and VBNC-like colonies.

### Phoenix colony emergence is eliminated by disruption of PA3626

**Figure 5:**
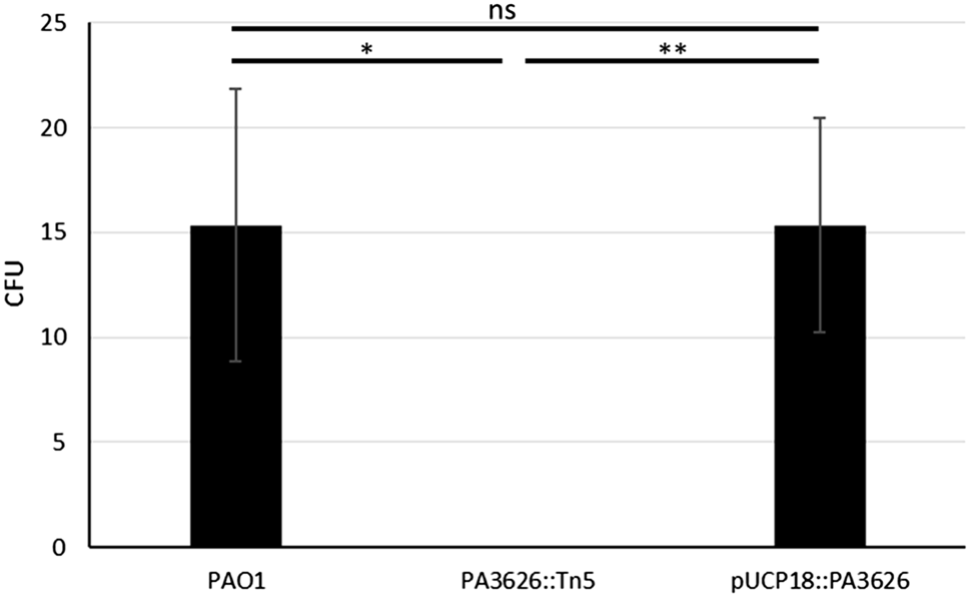
Complementation of PA3626 shows rescue of the phoenix phenotype. Phoenix colony counts were obtained for wild-type *P. aeruginosa* PAO1, the PA3626 transposon mutant, and the PA3626 complementation strain. After complementation of the transposon mutant, phoenix colony counts returned to wild-type levels, further confirming the importance of the tRNA pseudouridine synthase D in the emergence of phoenix colonies. *p < 0.05, **p < 0.01, n = 3.

Transposon mutants, from the Colin Manoil^15^ *P. aeruginosa* transposon mutant library, for each DEG were screened for changes in the frequency of phoenix colony emergence. Nine DEGs did not have a corresponding transposon mutant available in the library for screening (Table 1). Due to the comprehensive nature of the Manoil transposon library, these nine genes are likely essential. Yet, the transposon mutant for PA3626 produced a complete lack of phoenix colony emergence when exposed to tobramycin (n = 6). PA3626 encodes for the pseudouridine synthase D (TruD)^16^. Complementation of the full PA3626 gene into the PA3626 transposon mutant, using a pUCP18:PA3626 plasmid, restored the frequency of phoenix colony formation to wild-type levels (Fig. 5), indicating the importance of TruD in phoenix colony emergence.

### Phoenix colonies do not emerge in response to alternative ribosomal targeting antibiotics

Since phoenix colonies appear to only emerge in the presence of aminoglycosides^9^, additional testing was performed using tetracycline, an inhibitor of tRNA binding to the P site of the ribosome, and chloramphenicol, an inhibitor of peptide elongation. Neither tetracycline nor chloramphenicol showed emergence of phoenix colonies within the ZOC, further indicating that phoenix colonies are an aminoglycoside specific phenomenon.

## Discussion

*Pseudomonas aeruginosa* is an important bacterial pathogen implicated in both superficial and life-threatening infections that has readily developed tolerance and resistance to multiple antibacterial drugs. In this study, both the genomes and transcriptomes of both phoenix colonies and VBNC-like colonies were examined. Whole genome sequencing with pooling of the samples was used to identify conserved driver mutations. While VBNC-like isolates display a phenotype different than that of wild-type *P. aeruginosa*, both phenotypes were genotypically identical. However, when compared to wild-type *P. aeruginosa*, phoenix colonies have a SNP in the PA4673 gene which is predicted to encode a GTP-binding protein similar to YchF in *S. pombe*^13,14^. This SNP leads to a modification of the protein structure in an external region of the gene and could possibly lead to a change in a binding site. GTP binding proteins, also known as G proteins, are molecular switches which hydrolyze GTP to GDP and are bound to the membrane to allow for downstream effects to be stimulated by extracellular stimuli^17^. A modification in the ability of a G protein to bind to a substrate may have a major impact to downstream effectors within the pathway and may allow phoenix colonies to transiently tolerate the presence of high concentrations of antibiotic.

Transcriptionally, both phoenix colonies and VBNC-like colonies were found to cluster separately from cells in the control lawn biofilm which had not been exposed to antibiotics, the edge of the ZOC, and the outer background lawn controls, while clustering well with each other. It is interesting that both the phoenix colonies and VBNC-like colonies are transcriptomically similar to each other despite appearing as two distinct phenotypes. It is possible that VBNC-like colonies could be a subset of phoenix colonies which lack the ability to be cultured due to a dependence on a metabolite in the initial environment from either the original biofilm lawn or surrounding variant colonies. It is also interesting to note that no significant metabolic pathway changes were found in phoenix colonies or VBNC-like colonies which could account for the antibiotic tolerance exhibited by these phenotypes. This contrasts the typical tolerance phenotypes found in *P. aeruginosa* including persister cells, small colony variants, and adaptive resistance colonies, which enter a state of dormancy, have growth defects, or are otherwise metabolically driven^10,18–22^.

Upon further examination of the DEGs associated with phoenix colonies, one gene was found that when screened showed a lack of phoenix colony emergence: PA3626, which encodes for tRNA pseudouridine synthase D (TruD). Tobramycin, the antibiotic associated with the emergence of phoenix colonies^9^, is an aminoglycoside. Aminoglycosides bind irreversibly to the 30S portion of the bacterial ribosome, leading to mismatching binding of tRNA to the mRNA due to a steric hindrance during translation^23^. Antibiotic resistance to aminoglycosides can be conferred through modification of the ribosomal target site^24^. Additionally, it has been shown that aminoglycosides also interact directly with tRNA and can lead to a loss of interaction between the T- and D- loops as well as unfolding of the D-stem^25,26^. TruD converts uracil-13 in the D-loop to pseudouridine which may influence the way that aminoglycosides are able to interact with the tRNA^27^. Knockout of TruD by transposon mutagenesis led to an elimination of phoenix colony emergence which was able to be rescued to wild-type levels by plasmid complementation. This finding is interesting as it leads us to believe that, in addition to the ribosome, tobramycin may also be interacting with tRNA leading to cell death. Phoenix colonies may then be modifying their tRNA with TruD to increase tRNA stability and allow for survival with exposure to tobramycin, although this is something that will need to be followed up on for confirmation. Additionally, there is conflict between the PA3626 expression levels (down-regulated) and the PA3626 knockout data which indicated that PA3626 is necessary for phoenix colony emergence. Further research and understanding of PA3626 and its target sites may help to clarify these conflicting results and provide insight to the mechanisms involved.

Further research, including epigenetic exploration, is needed to come to a complete understanding of both the phoenix colonies and VBNC-like colonies. By understanding how these and other bacterial phenotypes are able to survive in the presence of antibiotics, we will be better prepared to control the rise of antibiotic tolerant and resistant infections.

## Materials and methods

### Bacterial strains and culture conditions

The bioluminescent strain *P. aeruginosa* Xen41 (Xenogen Corp., USA) was used for the imaging portion of this study (Fig. 1). *P. aeruginosa* Xen41 contains a metabolically driven lux operon which allows for visualization using IVIS and indicates the level of metabolic activity of the bacterial lawn and colonies, as an increase in luminescence correlates with an increase in bacterial metabolic activity. *P. aeruginosa* PAO1^15^, the parent strain for *P. aeruginosa* Xen41, was also used in this study. Stock culture plates were prepared by streaking from glycerol stock cultures which were stored at − 80 °C onto fresh Luria–Bertani (LB) agar plates. Streaked plates were incubated at 37 °C for 24 h. Individual colonies were isolated and transferred to 20 mL of LB broth. Broth cultures were incubated overnight in a shaking incubator at 37 °C and 200 rpm.

### Preparation of tobramycin loaded calcium sulfate beads

240 mg of tobramycin (Sigma-Aldrich) per 20 g of CaSO_4_ hemi-hydrate (Sigma-Aldrich) was used^28^. After mixing of the two powders, sterile water was mixed in to produce a thick paste. The paste was spread into silicone molds (Biocomposites Ltd.) to form 4.5 mm diameter, hemispherical beads. The beads were allowed to dry at room temperature overnight before being removed from the mold and stored at 4 °C. Additionally, beads containing tetracycline (960 mg/20 g CaSO_4_) and chloramphenicol (3.8 g/20 g CaSO_4_) were made.

### Preparation of phoenix and VBNC-like colonies of *P. aeruginosa*

Phoenix colonies were obtained as per Sindeldecker et al.^9^. Briefly, an overnight culture of *P. aeruginosa* PAO1 was diluted to an OD_600_ of 0.1 using sterile LB broth. One hundred µL of the diluted culture was spread onto LB agar in 100 mm diameter, polystyrene plates (Fisher Scientific, USA). The plates were incubated at 37 °C with 5% CO_2_ for 24 h in a humidified incubator (Heracell 150i, Thermo Scientific) to allow a lawn biofilm to develop. A tobramycin-loaded calcium sulfate bead was placed in the center of the plate and pushed into the agar using sterile forceps. The plates were then incubated further at 37 °C with 5% CO_2_ for an additional 96 h. Plates were checked daily for the appearance of a zone of clearance (ZOC) as well as colonies emerging within this zone. Additionally, 72 h lawn biofilms were prepared in the same fashion but without tobramycin beads for use as a control lawn.

### Isolation of samples to be used for whole genome sequencing

Phoenix colonies, VBNC-like colonies, and wild-type *P. aeruginosa* PAO1 colonies were isolated for whole genome sequencing. In short, phoenix colony plates were generated as above. After the emergence of the variant colonies on three replicate plates, 96 colonies from each plate were isolated into aliquots of 100 µL of PBS and stored at − 20 °C. Before placing the samples at − 20 °C, 5 µL was used to inoculate 200 µL of LB broth containing 5 µg/mL of tobramycin in a well of a 96-well plate (Corning, Sigma-Aldrich). An additional 5 µL was added to a corresponding well containing 200 µL of LB broth. The plates were incubated for 96 h at 37 °C with 5% CO_2_. After incubation, turbidity in the wells was visually compared. Variants which showed a lack of growth in the LB broth as well as the corresponding well of LB broth containing tobramycin were defined as VBNC-like colonies. Phoenix colonies were defined as those showing a lack of growth in the LB broth containing tobramycin while having growth in the LB only. An overnight broth culture of *P. aeruginosa* PAO1 was also grown as above to provide a wild-type sample for DNA extraction.

### Determination of variant colony antibiotic susceptibility

Isolates which grew from the previous section were subjected to a Kirby–Bauer assay to confirm whether they were resistant or susceptible to tobramycin. Each isolate was diluted to an OD_600_ of 0.1. 100 µL of each diluted culture was spread onto LB agar plates. A sterile, filter paper disk was placed in the center of each plate and 10 µg of a tobramycin solution (1 mg/mL in PBS) was placed on each disk. The plates were incubated at 37 °C with 5% CO_2_ for 24 h. After incubation, the diameter of the zone of inhibition was measured and compared to both WT *P. aeruginosa* PAO1 as well as the Clinical and Laboratory Standards Institute (CLSI) guidelines to determine if they were resistant or susceptible (phoenix colonies). Four phoenix colonies and four VBNC-like colonies were randomly selected for RNA extraction.

### Whole genome sequencing

DNA extraction was performed for each of the isolated samples using the GenElute™ Bacterial Genomic DNA kit (Sigma-Aldrich). After gDNA extraction, samples were pooled to obtain samples of 400 ng gDNA to identify conserved driver mutations. Library preparation and barcoding was completed using the Rapid Barcoding Kit (Nanopore). A Nanopore MinION Sequencer was used to complete bacterial whole genome sequencing using default settings. After sequencing, quality control was performed using Epi2ME (Nanopore). Reads were trimmed using Porechop^29^ and aligned to a reference *P. aeruginosa* PAO1 genome (GCF_000006765.1^30^) using graphmap^31^. Reads were sorted and indexed using samtools^32^. Structural variants were identified using sniffles^33^ and SNPs were identified using BCFtools^34^. SNPs were selected by a quality score > 20.

### Isolation of samples to be used for RNAseq

Once phoenix colony plates were prepared, samples were taken for use in RNA sequencing. Seventy-two hours post tobramycin bead placement, triplicate samples were taken from the lawn at the edge of the ZOC where variant colonies including phoenix colonies, VBNC-like colonies, and resistant colonies would be visible on the following day. The edge samples likely contained precursors to the variant colonies, WT cells, and dead and dying cells. Ninety-six hours post tobramycin bead placement, triplicate samples from the outer background lawn were taken and variant colonies which had emerged in the ZOC were isolated for phenotype characterization (phoenix colonies, VBNC-like colonies, or resistant mutants). Additionally, triplicate samples were taken from bacterial lawns grown for 72 h that had not been exposed to antibiotics. All samples were placed into 100 µL of RNAlater (Ambion) and stored at − 20 °C to protect the RNA from degradation. For variant colony isolates, a 5 µL sample was taken and used to inoculate 15 mL of sterile LB broth and incubated at 37 °C with 200 rpm shaking overnight. Cultures which did not grow were deemed to be VBNC-like colonies. Cultures which did grow were further examined to determine their level of susceptibility to tobramycin.

### RNA sequencing

RNA extraction was performed for each sample using the Direct-zol RNA Miniprep Kit (Zymo), including DNaseI treatment for DNA removal. RNA quantity was measured using Qubit 3.0. In order to remove ribosomal RNA, the RiboZero Bacteria Kit (Illumina) was used. Barcoded RNAseq libraries were generated using the ScriptSeq™ v2 Kit (Illumina) and 38 fmol of RNA was diluted to a final concentration of 1.9 nM in resuspension buffer using equal amounts of RNA from each sample for normalization. Samples were sequenced using paired-end (2 × 150 bp) sequencing on an Illumina HiSeq 4000 device. Sequencing reads were trimmed using Trimmomatic v0.38^35^, were quality controlled using FastQC v0.11.8^36^, and mapped to the reference genome for *P. aeruginosa* PAO1 (GCF_000006765.1^30^) using STAR v2.7.10a^37^. After read mapping, sequences were visualized to ensure adequate genome coverage using IGV (v2.4.13^38^). Transcript counts were obtained using HTseq v0.12.4^39^ and were analyzed for differential gene expression using the R package edgeR (v3.24.3^40^). Multi-dimensional scaling plots were generated using the plotMDS function within edgeR. Volcano plots were generated using the ggplot2 function in R^41^. Genes found to be differentially expressed when comparing phoenix colony and VBNC-like colony samples to 72 h unexposed lawn samples were submitted to DAVID 6.8^42,43^, KEGG^44^, and Gene Ontology^45,46^ for analysis.

### Screening DEG transposon mutants

Transposon mutants (obtained from the lab of Dr. Colin Manoil^15^) corresponding to each of the identified differentially expressed genes were screened in triplicate replicates for their ability to produce phoenix colonies in the same fashion as above. Phoenix colonies were distinguished using replica plating as per Sindeldecker et al.^9^. Briefly, a sterile, cotton velveteen square (150 × 150 mm) was draped over a PVC replica plating block and held in place using an aluminum ring. The tobramycin bead was removed from the plate using a sterile, plastic loop. The plate was marked to indicate a 12 o’clock position and gently placed on the velveteen square. The plate was gently tapped down before being removed from the replica plater. A fresh, sterile LB agar plate containing 5 μg/mL of tobramycin and marked at the 12 o’clock position was placed on the velveteen square and tapped down in the same fashion before being removed. A fresh, sterile LB agar plate marked at the 12 o’clock position was placed on the velveteen square and tapped down before being removed. Replica plates were incubated for 24 h at 37 °C with 5% CO_2_. After incubation, the colony pattern was compared and colonies which appeared on the LB agar replica plate but not on the LB agar replica plate containing tobramycin were determined to be phoenix colonies. Transposon mutant phoenix colony counts were obtained and compared to control *P. aeruginosa* PAO1 counts. The transposon mutant for PA3626 showed no emergence of phoenix colonies, and a control was performed to ensure that this lack of emergence was not due to a growth defect for the mutant. Both WT *P. aeruginosa* PAO1 and the PA3626 transposon mutant were plated and incubated at 37 °C with 5% CO_2_ for 24 h. After incubation, 1 cm × 1 cm areas were isolated and a dilution series was created, plated, and incubated at 37 °C with 5% CO_2_ for 24 h. CFU counts were obtained for each culture (3.0 × 10^8^ CFU/cm^2^ for WT and 7.0 × 10^8^ CFU/cm^2^ for the mutant) and compared (p = 0.34).

### Creation of a PA3626 complementation plasmid

*Pseudomonas aeruginosa* PAO1 gDNA was extracted using the GenElute™ Bacterial Genomic DNA kit. After extraction, PCR was performed to amplify the PA3626 gene using Phusion Taq polymerase (New England Biolabs) and forward (5′-GCGCGGATCCATGAGCGTTCTCGGCGAA) and reverse (5′-GCGCTCTAGATCAGTATGCGCATGGGTT) primers (Integrated DNA Technologies). After amplification, the PA3626 product was run on an agarose gel and extracted using the GenElute™ Gel Extraction kit. A DNA digestion was completed using the restriction enzymes BamHI and XbaI (New England Biolabs) on the PA3626 product and pUCP18 plasmid. DNA ligation was completed using the T4 DNA ligase (Thermo Fisher) and a 7:1 ratio of insert to plasmid. After ligation, the plasmid was electroporated into *P. aeruginosa* PAO1 PA3626::Tn5. Briefly, 1 mL of an overnight culture of PAO1 PA3626::Tn5 was centrifuged at max speed in a microcentrifuge for 1 min. The pellet was washed three times in 10% sucrose in sterile water before being resuspended in 100 µL of 10% sucrose. 5 µL of the ligated plasmid was added and the mixture was transferred to a 2 mm electroporation cuvette. The cuvette was electroporated at 25 µF, 200 Ω, and 2.5 kV. 1 mL of sterile LB was added to the cuvette and the culture was transferred to a 1.5 mL tube and incubated at 37 °C with 200 rpm shaking for 1 h. After incubation, the culture was spread on LB agar containing 100 µg/mL of carbenicillin and incubated overnight at 37 °C with 5% CO_2_. Isolates were cultured and plasmids were extracted using the Qiagen Spin Miniprep kit. Plasmids were digested using BamHI and XbaI restriction enzymes, and an agarose gel was run to confirm the appropriately sized bands were present.

### Screening complementation strain for phoenix colony emergence

The PA3626 complementation strain was plated and exposed to 1 mg of tobramycin as above. After variant colony emergence, plates were replica plated onto fresh LB agar and LB agar containing 5 µg/mL of tobramycin. Replica plates were incubated for 24 h at 37 °C with 5% CO_2_. After incubation, plates were compared to each other and colonies which grew on the original plate and LB only replica plate but did not grow on the replica plate containing tobramycin were counted as phoenix colonies.

### Protein modeling

Amino acid sequences for wild-type PA4673 and PA4673 containing the identified SNP were submitted for protein structure and functional modeling to Phyre2 on an internal development server^12^ using the intensive modeling mode. Models were visualized using RasMol^47^. Additionally, amino acid sequences for both wild-type and mutant PA4673 were submitted to the ExPASy server^48^.

### Statistical analysis

All experiments were performed with a minimum of triplicate technical replicates. ANOVA analysis was completed in edgeR for differentially expressed genes with significance set at a q-value of 0.05. ANOVA analysis was completed by GraphPad Prism (v8.2.1) for all other studies with a p-value of 0.05 being considered significant.

### Bioluminescence imaging

Bioluminescence imaging was performed using an in vitro imaging system (IVIS). Plates were exposed for thirty second exposures to obtain images. A pseudo-color heatmap was applied to aid in visualization.

## Data Availability

Raw data from RNA sequencing along with processed gene counts for each sample are available through the Gene Expression Omnibus, accession GSE199323. Raw sequencing reads from whole genome sequencing are available through the Sequence Read Archive, accession PRJNA820154.
